# Supplementary material for: Modelling Skylarks (Alauda arvensis) to Predict Impacts of Changes in Land Management and Policy: Development and Testing of an Agent-Based Model
Source: PLoS One. 2013 Jun 6;8(6):e65803. doi: 10.1371/journal.pone.0065803 (PMC3675089; doi:10.1371/journal.pone.0065803)
Supplement: Supporting Information S4 — The skylark ODdox as a zipped archive. (ZIP) [file pone.0065803.s004.zip › Skylark_ODdox/class_skylark___population___manager.html]

ALMaSS Skylark ODdox: Skylark\_Population\_Manager Class Reference


ALMaSS Skylark ODdox
 2.0


- Main Page
- Related Pages
- Classes
- Files

- Class List
- Class Index
- Class Hierarchy
- Class Members

Public Member Functions |
Public Attributes |
Protected Member Functions |
Protected Attributes

Skylark\_Population\_Manager Class Reference

`#include <skylarks_all.h>`

List of all members.

|  |  |
| --- | --- |
| Public Member Functions | |
| void | AddStriglingMort (int lifestage) |
| virtual void | BreedingPairsOutput (int Time) |
| virtual void | BreedingSuccessProbeOutput (double, int, int, int, int, int, int, int) |
| void | CreateObjects (int ob\_type, TAnimal \*pTAo, void \*null, Skylark\_struct \*data, int number) |
| void | FledgelingProbeOutput (int Total, int Time) |
| void | incNoChickDeaths () |
| void | incNoFledgeDeaths () |
| void | incNoPestEffects () |
| void | incTotalEggs (int eggs) |
| void | incTotalNestlings () |
| void | incTotalPrefledgelings () |
| virtual void | Init (void) |
| bool | IsBadWeather () |
| bool | OpenTheBreedingPairsProbe () |
| virtual bool | OpenTheBreedingSuccessProbe () |
| bool | OpenTheFledgelingProbe () |
| void | SetF\_Mig\_Mort (int m) |
| void | SetM\_Mig\_Mort (int m) |
|  | Skylark\_Population\_Manager (Landscape \*L) |
| double | SupplyEMi () |
| int | SupplyF\_Mig\_Mort () |
| int | SupplyM\_Mig\_Mort () |
| int | SupplyNoTerritories () |
| int | TheBreedingFemalesProbe (int ProbeNo) |
| virtual int | TheBreedingSuccessProbe (int &BreedingFemales, int &YoungOfTheYear, int &TotalPop, int &TotalFemales, int &TotalMales, int &BreedingAttempts) |
| virtual void | TheCIPEGridOutputProbe () |
| int | TheFledgelingProbe () |
| virtual void | TheNWordOutputProbe () |
| virtual void | TheRipleysOutputProbe (FILE \*a\_prb) |
| int | TheSkylarkTerrsSupply\_quality (int) |
| int | TheSkylarkTerrsSupply\_size (int) |
| int | TheSkylarkTerrsSupply\_x (int) |
| int | TheSkylarkTerrsSupply\_y (int) |
| void | WriteSKPOM1 (int n, int n2) |
| void | WriteSKPOM2 (int n, int n2) |
| virtual | ~Skylark\_Population\_Manager (void) |
| Public Member Functions inherited from Population\_Manager | |
| bool | BeginningOfMonth () |
| virtual void | Catastrophe (int) |
| bool | CheckXY (int l, int i) |
|  | Debug method to test for out of bounds coordinates. |
| void | CloseTheCIPEGridOutputProbe () |
| void | CloseTheMonthlyRipleysOutputProbe () |
| void | CloseTheReallyBigOutputProbe () |
| void | CloseTheRipleysOutputProbe () |
| virtual void | DisplayLocations () |
| TAnimal \* | FindClosest (int x, int y, unsigned Type) |
| virtual void | GeneticsResultsOutput (FILE \*, unsigned) |
| virtual void | ImpactedProbe () |
| void | ImpactProbeReport (int a\_Time) |
| bool | IsLast (unsigned listindex) |
| void | LamdaBirth (int x, int y) |
| void | LamdaBirth (int x, int y, int z) |
| void | LamdaClear () |
| void | LamdaDeath (int x, int y) |
| void | LamdaDumpOutput () |
| void | LOG (const char \*fname) |
| bool | OpenTheCIPEGridOutputProbe () |
| bool | OpenTheMonthlyRipleysOutputProbe () |
| bool | OpenTheReallyBigProbe () |
| bool | OpenTheRipleysOutputProbe () |
|  | Population\_Manager (Landscape \*L) |
| virtual float | Probe (int ListIndex, probe\_data \*p\_TheProbe) |
| int | ProbeFileInput (char \*p\_Filename, int p\_ProbeNo) |
| char \* | ProbeReport (int a\_time) |
| char \* | ProbeReportTimed (int a\_time) |
| virtual void | Run (int NoTSteps) |
| void | SetNoProbes (int a\_pn) |
| char \* | SpeciesSpecificReporting (int a\_species, int a\_time) |
| virtual int | SupplyCovPosx (int) |
| virtual int | SupplyCovPosy (int) |
| unsigned | SupplyListIndexSize () |
| const char \* | SupplyListName (int i) |
| int | SupplyListNameLength () |
| unsigned | SupplyListSize (unsigned listindex) |
| virtual void | SupplyLocXY (unsigned listindex, unsigned j, int &x, int &y) |
| virtual int | SupplyPegPosx (int) |
| virtual int | SupplyPegPosy (int) |
| int | SupplySimH () |
| int | SupplySimW () |
| int | SupplyState (unsigned listindex, unsigned j) |
| IntArray100 \* | SupplyStateList () |
| const char \* | SupplyStateNames (int i) |
| unsigned | SupplyStateNamesLength () |
| int | SupplyStepSize () |
| virtual void | TheGeneticProbe (unsigned, int, unsigned &) |
| virtual void | TheReallyBigOutputProbe () |
| virtual | ~Population\_Manager (void) |

|  |  |
| --- | --- |
| Public Attributes | |
| SkTerritories \* | TheSkylarkTerrs |
| int | VegTypeFledgelings [100] |
| Public Attributes inherited from Population\_Manager | |
| AlleleFreq \* | AlFreq |
| unsigned | Counts [10][100] |
| int | IndexArrayX [5][10000] |
| MyDialog \* | m\_MainForm |
| char | m\_SimulationName [255] |
| Landscape \* | m\_TheLandscape |
| bool | ProbesSet |
| int | SimH |
| unsigned | SimHH |
| int | SimW |
| unsigned | SimWH |
| probe\_data \* | TheProbe [100] |

|  |  |
| --- | --- |
| Protected Member Functions | |
| virtual void | Catastrophe () |
| virtual void | DoFirst () |
| void | LoadParameters () |
| float | ProbePOM (int ListIndex, probe\_data \*p\_TheProbe) |
|  | Modified probe for POM Output. |
| void | ProbeReportPOM (int a\_time) |
| virtual void | ReHouse () |
| Protected Member Functions inherited from Population\_Manager | |
| virtual void | DoAfter () |
| virtual void | DoAlmostLast () |
| virtual void | DoBefore () |
| virtual void | DoLast () |
| void | NWordOutput () |
|  | The output section for the NWord probe.  . |
| void | Shuffle (unsigned Type) |
| void | Shuffle\_or\_Sort (unsigned Type) |
| void | SortState (unsigned Type) |
| void | SortStateR (unsigned Type) |
| void | SortX (unsigned Type) |
| void | SortXIndex (unsigned Type) |
| void | SortY (unsigned Type) |
| virtual bool | StepFinished () |

|  |  |
| --- | --- |
| Protected Attributes | |
| double | EMi |
| int | F\_Mig\_Mort |
| bool | m\_IsBadWeather |
| int | M\_Mig\_Mort |
| int | m\_NoChickDeaths |
| int | m\_NoFledgeDeaths |
| int | m\_NoPestEffects |
| int | m\_StriglingMort [4] |
| int | m\_TotalEggs |
| int | m\_TotalNestlings |
| int | m\_TotalPrefledgelings |
| FILE \* | SKPOM1 |
| FILE \* | SKPOM2 |
| Protected Attributes inherited from Population\_Manager | |
| unsigned | BeforeStepActions [10] |
| FILE \* | CIPEGridOutputPrb |
| FILE \* | CIPEGridOutputPrbB |
| long int | lamdagrid [2][257][257] |
| FILE \* | m\_AlleleFreqsFile |
| int | m\_catastrophestartyear |
| int \* | m\_cgridcount |
| int | m\_cgridcountwidth |
| int | m\_cipegridsize |
| FILE \* | m\_EasyPopRes |
| FILE \* | m\_GeneticsFile |
| int \* | m\_gridcount [4] |
| int | m\_gridcountsize [4] |
| int | m\_gridcountwidth [4] |
| unsigned | m\_ListNameLength |
| const char \* | m\_ListNames [10] |
| int | m\_NoProbes |
| int | m\_StepSize |
| FILE \* | NWordOutputPrb |
| FILE \* | ReallyBigOutputPrb |
| FILE \* | RipleysOutputPrb |
| FILE \* | RipleysOutputPrb1 |
| FILE \* | RipleysOutputPrb10 |
| FILE \* | RipleysOutputPrb11 |
| FILE \* | RipleysOutputPrb12 |
| FILE \* | RipleysOutputPrb2 |
| FILE \* | RipleysOutputPrb3 |
| FILE \* | RipleysOutputPrb4 |
| FILE \* | RipleysOutputPrb5 |
| FILE \* | RipleysOutputPrb6 |
| FILE \* | RipleysOutputPrb7 |
| FILE \* | RipleysOutputPrb8 |
| FILE \* | RipleysOutputPrb9 |
| IntArray100 | StateList |
| const char \* | StateNames [100] |
| unsigned | StateNamesLength |
| FILE \* | TestFile |
| FILE \* | TestFile2 |
| vector< TListOfAnimals > | TheArray |

---

## Constructor & Destructor Documentation

|  |  |  |  |  |  |
| --- | --- | --- | --- | --- | --- |
| Skylark\_Population\_Manager::Skylark\_Population\_Manager | ( | Landscape \* | *L* | ) |  |

References F\_Mig\_Mort, Init(), JuvenileReturnMort, M\_Mig\_Mort, Population\_Manager::TheArray, TheSkylarkTerrs, and CfgInt::value().

: Population\_Manager( L ) {

// Five lists are needed so need to remove 5 of the ten default arrays

// Clutches,Nestlings,PreFledgelings,Males,Females

for ( int i = 0; i < 5; i++ ) {

TheArray.pop\_back();

}

M\_Mig\_Mort = 0;

F\_Mig\_Mort = 0;

//

/\*

F\_Mig\_Mort\_min = cfg\_femreturnmortmin.value();

F\_Mig\_Mort\_max = cfg\_femreturnmortmax.value();

M\_Mig\_Mort\_min = cfg\_malereturnmortmin.value();

M\_Mig\_Mort\_max = cfg\_malereturnmortmax.value();

\*/

//

JuvenileReturnMort = cfg\_juvreturnmort.value();

//SkYoungWetDay = cfg\_skyoungwetday.value();

TheSkylarkTerrs = new SkTerritories( L );

#ifdef \_\_CJTDebug\_10

refnum = 0;

#endif

Init();

}

|  |  |  |  |  |  |
| --- | --- | --- | --- | --- | --- |
| Skylark\_Population\_Manager::~Skylark\_Population\_Manager | ( | void |  | ) |  |

virtual

References SKPOM1, SKPOM2, and TheSkylarkTerrs.

{

// delete all lists

delete TheSkylarkTerrs;

#ifdef \_\_SKPOM

fclose(SKPOM1);

fclose(SKPOM2);

#endif

}

---

## Member Function Documentation

|  |  |  |  |  |  |
| --- | --- | --- | --- | --- | --- |
| void Skylark\_Population\_Manager::AddStriglingMort | ( | int | *lifestage* | ) |  |

inline

{

m\_StriglingMort[lifestage] ++;

}

|  |  |  |  |  |  |
| --- | --- | --- | --- | --- | --- |
| void Skylark\_Population\_Manager::BreedingPairsOutput | ( | int | *Time* | ) |  |

virtual

Reimplemented from Population\_Manager.

References g\_msg, Skylark\_Base::m\_CurrentSkState, Skylark\_Adult::Paired, TAnimal::Supply\_m\_Location\_x(), TAnimal::Supply\_m\_Location\_y(), Landscape::SupplyFarmOwner(), Population\_Manager::TheArray, toss\_BuildingUpResources, toss\_EggHatching, toss\_FCaringForYoung, toss\_Incubating, toss\_Laying, toss\_MakingNest, toss\_PreparingForBreeding, MapErrorMsg::Warn(), and WARN\_FILE.

{

FILE \* MyFile = fopen("BreedingPairs.txt", "a" );

if ( !MyFile ) {

g\_msg->Warn( WARN\_FILE, "Skylark\_Population\_Manager::BreedingPairsOutput():",

"Cannot open file for append: BreedingPairs.txt" );

exit( 0 );

}

int no=0;

Skylark\_Female \* FS;

int szf = (int)TheArray[ 4 ].size();

int szm = (int)TheArray[ 3 ].size();

for ( int j = 0; j < szf; j++ ) {

FS = dynamic\_cast < Skylark\_Female \* > ( TheArray[ 4 ] [ j ] );

if (FS->Paired) {

// Below is a POM test for farm owner 50 - an identifier used to select a single experimental field or set of fields in the polyref file.

if (g\_land->SupplyFarmOwner(FS->Supply\_m\_Location\_x(), FS->Supply\_m\_Location\_y()) == 50){

TTypesOfSkState behav = FS->m\_CurrentSkState;

switch(behav) {

case toss\_BuildingUpResources:

case toss\_PreparingForBreeding:

case toss\_MakingNest:

case toss\_Laying:

case toss\_EggHatching:

case toss\_Incubating:

case toss\_FCaringForYoung:

no++;

// g\_MainForm->m\_aMap->Spot( 3, FS->Supply\_m\_Location\_x(), FS->Supply\_m\_Location\_y() );

break;

default:

;

}

}

}

}

fprintf( MyFile, "%d\t%d\t%d\t%d\n", time, no, szf, szm );

fclose( MyFile );

}

|  |  |  |  |
| --- | --- | --- | --- |
| void Skylark\_Population\_Manager::BreedingSuccessProbeOutput | ( | double | *No*, |
|  |  | int | *BreedingFemales*, |
|  |  | int | *YoungOfTheYear*, |
|  |  | int | *TotalPop*, |
|  |  | int | *TotalFemales*, |
|  |  | int | *TotalMales*, |
|  |  | int | *time*, |
|  |  | int | *BreedingAttempts* |
|  | ) |  |  |

virtual

Reimplemented from Population\_Manager.

References g\_msg, m\_StriglingMort, m\_TotalEggs, m\_TotalNestlings, m\_TotalPrefledgelings, MapErrorMsg::Warn(), and WARN\_FILE.

{

double BreedingSuccess, HatchSuccess, FledgeSuccess;

FILE \* MyFile = fopen("BreedingSuccess.txt", "a" );

if ( !MyFile ) {

g\_msg->Warn( WARN\_FILE, "Skylark\_Population\_Manager::BreedingAttemptsOutput():",

"Cannot open file for append: BreedingAttempts.txt" );

exit( 0 );

}

// Do some calculations

double floaters = ( TotalPop - ( YoungOfTheYear + ( BreedingFemales \* 2 ) ) ) / ( double )( TotalPop - YoungOfTheYear );

HatchSuccess = m\_TotalNestlings / ( double )( m\_TotalEggs + 1 );

FledgeSuccess = m\_TotalPrefledgelings / ( double )( m\_TotalNestlings + 1 );

BreedingSuccess = m\_TotalPrefledgelings / ( double )( m\_TotalEggs + 1 );

// Print the results and data

fprintf( MyFile, "%d\t%d\t%d\t%d\t%d\t%d\t%d\t%g\t%g\t%g\t%g\t%g\t\n", time, BreedingFemales, BreedingAttempts, YoungOfTheYear, TotalPop,

TotalFemales, TotalMales, floaters, No, HatchSuccess, FledgeSuccess, BreedingSuccess );

//fprintf( MyFile, "%i\t%i\t%i\t%i\n", m\_StriglingMort[ 0 ], m\_StriglingMort[ 1 ],

// m\_StriglingMort[ 2 ], m\_StriglingMort[ 3 ] );

for ( int h = 0; h < 4; h++ ) m\_StriglingMort[ h ] = 0;

fclose( MyFile );

// Zero any counters

m\_TotalEggs = 0;

m\_TotalNestlings = 0;

m\_TotalPrefledgelings = 0;

}

|  |  |  |  |  |  |
| --- | --- | --- | --- | --- | --- |
| void Skylark\_Population\_Manager::Catastrophe | ( | void |  | ) |  |

protectedvirtual

Reimplemented from Population\_Manager.

References Skylark\_Adult::CopyMyself(), TAnimal::Dying(), Population\_Manager::m\_catastrophestartyear, Population\_Manager::m\_TheLandscape, Skylark\_Female::MyMate, Skylark\_Male::MyMate, Skylark\_Female::OnMateDying(), Skylark\_Male::OnMateDying(), random(), Landscape::SupplyDayInYear(), Landscape::SupplyYearNumber(), Population\_Manager::TheArray, and CfgInt::value().

{

// This version simply alters populations on 1st January - it is very dangerous to

// add individuals in many of the models so beware!!!!

// First do we have the right day?

int today = m\_TheLandscape->SupplyDayInYear();

if (today!=1) return;

// First do we have the right year?

int year = m\_TheLandscape->SupplyYearNumber()-m\_catastrophestartyear;

if (year%cfg\_pm\_eventfrequency.value()!=0) return;

Skylark\_Male\* MS = NULL; // assignment to get rid of warning

Skylark\_Female\* FS = NULL;

Skylark\_Adult\* AS = NULL;

// Now if the % decrease is higher or lower than 100 we need to do different things

int esize=cfg\_pm\_eventsize.value();

if (esize<100) {

unsigned size2;

size2 = (int)TheArray[ 3 ].size();

for ( unsigned j = 0; j < size2; j++ ) {

if (random(100) > esize) {

MS = dynamic\_cast < Skylark\_Male \* > ( TheArray[ 3 ] [ j ] );

if (MS->MyMate!=NULL) MS->MyMate->OnMateDying();

MS->Dying(); // Kill it

}

}

size2 = (int)TheArray[ 4 ].size();

for ( unsigned j = 0; j < size2; j++ ) {

if (random(100) > esize) {

FS = dynamic\_cast < Skylark\_Female \* > ( TheArray[ 4 ] [ j ] );

if (FS->MyMate!=NULL) FS->MyMate->OnMateDying();

FS->Dying(); // Kill it

}

}

}

else if (esize>100) {

// This is a tricky thing to do because we need to duplicate birds, but dare not mess

// mate pointers etc up.

// This also requires a copy method in the target birds

// esize also needs translating 120 = 20%, 200 = 100%

if (esize<200) {

esize-=100;

for (unsigned i=3; i<5; i++) {

unsigned size2;

size2 = (int)TheArray[ i ].size();

for ( unsigned j = 0; j < size2; j++ ) {

if (random(100) < esize) {

AS = dynamic\_cast < Skylark\_Adult \* > ( TheArray[ i ] [ j ] );

AS->CopyMyself(i); // Duplicate it

}

}

}

} else {

esize-=100;

esize/=100; // this will throw away fractional parts so will get 1, 2, 3 from 200, 350 400

for (unsigned i=3; i<5; i++) {

unsigned size2;

size2 = (int)TheArray[ i ].size();

for ( unsigned j = 0; j < size2; j++ ) {

for ( int e=0; e<esize; e++) {

AS = dynamic\_cast < Skylark\_Adult \* > ( TheArray[ i ] [ j ] );

AS->CopyMyself(i); // Duplicate it

}

}

}

}

}

else return; // No change so do nothing

}

|  |  |  |  |
| --- | --- | --- | --- |
| void Skylark\_Population\_Manager::CreateObjects | ( | int | *ob\_type*, |
|  |  | TAnimal \* | *pTAo*, |
|  |  | void \* | *null*, |
|  |  | Skylark\_struct \* | *data*, |
|  |  | int | *number* |
|  | ) |  |  |

References PreFledgeling\_struct::age, AdultSkylark\_struct::age, Skylark\_struct::bx, Skylark\_struct::by, Nestling\_struct::Dad, PreFledgeling\_struct::Dad, Skylark\_struct::L, m\_TotalNestlings, m\_TotalPrefledgelings, Skylark\_struct::mh, skClutch\_struct::Mum, skClutch\_struct::No, Skylark\_Male::OnAddNestling(), Skylark\_Male::OnAddPreFledgeling(), Skylark\_Female::OnSetMyClutch(), PreFledgeling\_struct::sex, PreFledgeling\_struct::size, AdultSkylark\_struct::size, Skylark\_struct::Terrs, Population\_Manager::TheArray, Skylark\_struct::x, and Skylark\_struct::y.

Referenced by Init(), Skylark\_Clutch::st\_Hatching(), Skylark\_Female::st\_Laying(), Skylark\_Nestling::st\_Maturing(), and Skylark\_PreFledgeling::st\_Maturing().

{

Skylark\_Clutch \* New\_Clutch;

Skylark\_Nestling \* New\_Nestling;

Skylark\_PreFledgeling \* New\_PreFledgeling;

Skylark\_Male \* New\_Male;

Skylark\_Female \* New\_Female;

skClutch\_struct \* cs;

Nestling\_struct \* ns;

PreFledgeling\_struct \* pfs;

AdultSkylark\_struct \* as;

bool aCopy=false;

if (number==-1) { aCopy=true; number=1; }

for ( int i = 0; i < number; i++ ) {

#ifdef \_\_CJTDebug\_10

refnum++;

#endif

if ( ob\_type == 0 ) {

cs = dynamic\_cast < skClutch\_struct \* > ( data );

New\_Clutch = new Skylark\_Clutch( cs->Mum, cs->Terrs, cs->L, cs->No, cs->x, cs->y, cs->mh, this );

TheArray[ ob\_type ].push\_back( New\_Clutch );

// Give mum the pointer to the clutch

cs->Mum->OnSetMyClutch( New\_Clutch );

#ifdef \_\_CJTDebug\_10

New\_Clutch->m\_Ref = refnum;

#endif

}

if ( ob\_type == 1 ) {

ns = dynamic\_cast < Nestling\_struct \* > ( data );

New\_Nestling = new Skylark\_Nestling( ns->x, ns->y, ns->Dad, ns->L, ns->Terrs, this, ns->bx, ns->by, ns->mh );

TheArray[ ob\_type ].push\_back( New\_Nestling );

// Give Dad a pointer to nestling

ns->Dad->OnAddNestling( New\_Nestling );

// Count this as a chick hatched

m\_TotalNestlings++;

#ifdef \_\_CJTDebug\_10

New\_Nestling->m\_Ref = refnum;

#endif

}

if ( ob\_type == 2 ) {

pfs = dynamic\_cast < PreFledgeling\_struct \* > ( data );

New\_PreFledgeling = new

Skylark\_PreFledgeling( pfs->x, pfs->y, pfs->L, pfs->Terrs, pfs->Dad, pfs->sex, pfs->size, pfs->age, this,

pfs->bx, pfs->by, pfs->mh );

TheArray[ ob\_type ].push\_back( New\_PreFledgeling );

if ( pfs->Dad ) pfs->Dad->OnAddPreFledgeling( New\_PreFledgeling, ( Skylark\_Nestling \* ) pTAo );

// Count this as a nest leaver

m\_TotalPrefledgelings++;

}

if ( ob\_type == 3 ) {

as = dynamic\_cast < AdultSkylark\_struct \* > ( data );

// Male

New\_Male = new Skylark\_Male( as->x, as->y, as->size, as->age, as->Terrs, as->L, this, as->bx, as->by, as->mh );

if (aCopy) New\_Male->SensibleCopy();

TheArray[ ob\_type ].push\_back( New\_Male );

#ifdef \_\_CJTDebug\_10

New\_Male->m\_Ref = refnum;

#endif

}

if ( ob\_type == 4 ) {

as = dynamic\_cast < AdultSkylark\_struct \* > ( data );

// Female

New\_Female = new Skylark\_Female( as->x, as->y, as->size, as->age, as->Terrs, as->L, this, as->bx, as->by, as->mh );

if (aCopy) New\_Female->SensibleCopy();

TheArray[ ob\_type ].push\_back( New\_Female );

#ifdef \_\_CJTDebug\_10

New\_Female->m\_Ref = refnum;

#endif

}

}

}

|  |  |  |  |  |  |
| --- | --- | --- | --- | --- | --- |
| void Skylark\_Population\_Manager::DoFirst | ( | void |  | ) |  |

protectedvirtual

Can be used in descendent classes

Reimplemented from Population\_Manager.

References Calendar::Date(), EMi, SkTerritories::EvaluateAllTerritories(), F\_Mig\_Mort, g\_date, g\_weather, Weather::GetRain(), Weather::GetTempPeriod(), Weather::GetWindPeriod(), January, m\_IsBadWeather, M\_Mig\_Mort, m\_NoChickDeaths, m\_NoFledgeDeaths, m\_NoPestEffects, m\_StriglingMort, Population\_Manager::m\_TheLandscape, SkTerritories::PreProcessLandscape2(), ProbeReportPOM(), Landscape::SupplyDayInYear(), Landscape::SupplyTemp(), TheSkylarkTerrs, SkTerritories::Tick(), and CfgInt::value().

{

//printf("Day %d Terrs: %d Occupied M/F %d/%d\n",g\_date->DayInYear(),TheSkylarkTerrs->SupplyNoTerritories(), TheSkylarkTerrs->SupplyNoMaleOccupied(), TheSkylarkTerrs->SupplyNoFemaleOccupied());

// Bad weather criteria - not used as of 24 June 2009 because removing this had no impacts on the observed POM testing.

if ((g\_weather->GetRain()>0.0) && (g\_weather->GetWindPeriod(g\_date->Date(),7)>45.0) && (g\_weather->GetTempPeriod(g\_date->Date(),7)<40.0) ) m\_IsBadWeather=true; else m\_IsBadWeather=false;

TheSkylarkTerrs->Tick();

double temp = m\_TheLandscape->SupplyTemp();

EMi = ( 31.2 - ( 0.44 \* temp ) );

int today = m\_TheLandscape->SupplyDayInYear();

if ( today == January ) {

for ( int h = 0; h < 4; h++ ) m\_StriglingMort[ h ] = 0;

TheSkylarkTerrs->PreProcessLandscape2( m\_TheLandscape );

// Now re-house any males that already had territories

//ReHouse(); // Only needed if PreProcessLandsape2 is called after arrival

// Determine this years mortality

F\_Mig\_Mort = cfg\_adultreturnmort.value(); //F\_Mig\_Mort\_min + random( F\_Mig\_Mort\_max - F\_Mig\_Mort\_min );

// F\_Mig\_Mort=50;

//assume that male and female mortality is equal

M\_Mig\_Mort = F\_Mig\_Mort;

//M\_Mig\_Mort = M\_Mig\_Mort\_min + random( M\_Mig\_Mort\_max - M\_Mig\_Mort\_min );

// F\_Mig\_Mort=F\_Mig\_Mort\_min+random(F\_Mig\_Mort\_max-F\_Mig\_Mort\_min);

// \*\*CJT\*\* testing code below

FILE\* MyFile=fopen( "PestEffects.txt", "a" );

fprintf( MyFile, "%d %d %d\n", m\_NoChickDeaths, m\_NoFledgeDeaths, m\_NoPestEffects );

fclose( MyFile );

m\_NoFledgeDeaths = 0;

m\_NoChickDeaths = 0;

m\_NoPestEffects = 0;

}

#ifdef \_\_SKPOM

// Skylark POM outputs

#ifdef \_\_KALONOSCRAPES

/\* USE FOR KALO & NO SCRAPES \*/

switch (today) {

case 70:

return ProbeReportPOM( today );

break;

case 80:

return ProbeReportPOM( today );

break;

case 89:

return ProbeReportPOM( today );

break;

case 99:

return ProbeReportPOM( today );

break;

case 115:

return ProbeReportPOM( today );

break;

case 123:

return ProbeReportPOM( today );

break;

case 130:

return ProbeReportPOM( today );

break;

case 140:

return ProbeReportPOM( today );

break;

case 147:

return ProbeReportPOM( today );

break;

case 153:

return ProbeReportPOM( today );

break;

case 160:

return ProbeReportPOM( today );

break;

case 168:

return ProbeReportPOM( today );

break;

case 177:

return ProbeReportPOM( today );

break;

case 185:

return ProbeReportPOM( today );

break;

case 192:

return ProbeReportPOM( today );

break;

case 200:

return ProbeReportPOM( today );

break;

case 209:

return ProbeReportPOM( today );

break;

default: ;

}

#else

#ifdef \_\_KALOSCRAPES

/\* USE FOR KALO & SCRAPES \*/

switch (today) {

case 67:

return ProbeReportPOM( today );

break;

case 70:

return ProbeReportPOM( today );

break;

case 80:

return ProbeReportPOM( today );

break;

case 89:

return ProbeReportPOM( today );

break;

case 99:

return ProbeReportPOM( today );

break;

case 113:

return ProbeReportPOM( today );

break;

case 125:

return ProbeReportPOM( today );

break;

case 130:

return ProbeReportPOM( today );

break;

case 131:

return ProbeReportPOM( today );

break;

case 140:

return ProbeReportPOM( today );

break;

case 147:

return ProbeReportPOM( today );

break;

case 153:

return ProbeReportPOM( today );

break;

case 162:

return ProbeReportPOM( today );

break;

case 169:

return ProbeReportPOM( today );

break;

case 178:

return ProbeReportPOM( today );

break;

case 183:

return ProbeReportPOM( today );

break;

default: ;

}

#else

switch (today) {

case 105:

return ProbeReportPOM( today );

break;

case 119:

return ProbeReportPOM( today );

break;

case 133:

return ProbeReportPOM( today );

break;

case 147:

return ProbeReportPOM( today );

break;

case 162:

return ProbeReportPOM( today );

break;

case 177:

return ProbeReportPOM( today );

break;

case 192:

return ProbeReportPOM( today );

break;

case 207:

return ProbeReportPOM( today );

break;

default: ;

}

#endif

#endif

#endif

//Sigh, everyday we need to update the territory qualities, but at least it only needs doing here!

TheSkylarkTerrs->EvaluateAllTerritories();

}

|  |  |  |  |
| --- | --- | --- | --- |
| void Skylark\_Population\_Manager::FledgelingProbeOutput | ( | int | *Total*, |
|  |  | int | *Time* |
|  | ) |  |  |

virtual

Reimplemented from Population\_Manager.

References g\_msg, Landscape::GetVegArea(), Population\_Manager::m\_TheLandscape, tov\_Undefined, VegTypeFledgelings, MapErrorMsg::Warn(), and WARN\_FILE.

{

FILE \* MyFile = fopen("SkFledgelingProbe.txt", "a" );

if ( !MyFile ) {

g\_msg->Warn( WARN\_FILE, "Skylark\_Population\_Manager::FledgelingProbeOutput():",

"Cannot open file for append: SkFledgelingProbe.txt" );

exit( 0 );

}

fprintf( MyFile, "%8d %6d", time, Total );

// Now get the areas

for ( int i = 0; i <= tov\_Undefined; i++ )

fprintf( MyFile, " %3d %5d %9.0f", i, VegTypeFledgelings[ i ], m\_TheLandscape->GetVegArea( i ) );

fprintf( MyFile, "\n" );

fclose( MyFile );

// Data for the FledgelingProbe needs clearing

for ( int i = 0; i < 100; i++ ) {

VegTypeFledgelings[ i ] = 0;

}

}

|  |  |  |  |  |
| --- | --- | --- | --- | --- |
| void Skylark\_Population\_Manager::incNoChickDeaths | ( |  | ) |  |

inline

{

m\_NoChickDeaths++;

}

|  |  |  |  |  |
| --- | --- | --- | --- | --- |
| void Skylark\_Population\_Manager::incNoFledgeDeaths | ( |  | ) |  |

inline

{

m\_NoFledgeDeaths++;

}

|  |  |  |  |  |
| --- | --- | --- | --- | --- |
| void Skylark\_Population\_Manager::incNoPestEffects | ( |  | ) |  |

inline

{

m\_NoPestEffects++;

}

|  |  |  |  |  |  |
| --- | --- | --- | --- | --- | --- |
| void Skylark\_Population\_Manager::incTotalEggs | ( | int | *eggs* | ) |  |

inline

{

m\_TotalEggs+=eggs;

}

|  |  |  |  |  |
| --- | --- | --- | --- | --- |
| void Skylark\_Population\_Manager::incTotalNestlings | ( |  | ) |  |

inline

{

m\_TotalNestlings++;

}

|  |  |  |  |  |
| --- | --- | --- | --- | --- |
| void Skylark\_Population\_Manager::incTotalPrefledgelings | ( |  | ) |  |

inline

{

m\_TotalPrefledgelings++;

}

|  |  |  |  |  |  |
| --- | --- | --- | --- | --- | --- |
| void Skylark\_Population\_Manager::Init | ( | void |  | ) |  |

virtual

References AdultSkylark\_struct::age, Population\_Manager::BeforeStepActions, Skylark\_struct::bx, Skylark\_struct::by, CreateObjects(), Skylark\_struct::L, LoadParameters(), Population\_Manager::m\_ListNameLength, Population\_Manager::m\_ListNames, m\_NoChickDeaths, m\_NoFledgeDeaths, m\_NoPestEffects, Population\_Manager::m\_SimulationName, Population\_Manager::m\_TheLandscape, m\_TotalEggs, m\_TotalNestlings, m\_TotalPrefledgelings, Skylark\_struct::mh, Population\_Manager::OpenTheCIPEGridOutputProbe(), Population\_Manager::OpenTheReallyBigProbe(), Population\_Manager::OpenTheRipleysOutputProbe(), random(), Population\_Manager::ReallyBigOutputPrb, AdultSkylark\_struct::sex, AdultSkylark\_struct::size, SKPOM1, SKPOM2, Population\_Manager::StateNames, Landscape::SupplySimAreaHeight(), Landscape::SupplySimAreaWidth(), Skylark\_struct::Terrs, TheSkylarkTerrs, toss\_AttractingAMate, toss\_BuildingUpResources, toss\_CDying, toss\_Destroy, toss\_Developing, toss\_EggHatching, toss\_FArriving, toss\_FCaringForYoung, toss\_FDying, toss\_FEmigrating, toss\_FFindingTerritory, toss\_FFloating, toss\_FFlocking, toss\_FImmigrating, toss\_FollowingMate, toss\_FTempLeavingArea, toss\_GivingUpTerritory, toss\_Hatching, toss\_Incubating, toss\_Initiation, toss\_Laying, toss\_MakingNest, toss\_MArriving, toss\_MCaringForYoung, toss\_MDying, toss\_MEmigrating, toss\_MFindingTerritory, toss\_MFloating, toss\_MFlocking, toss\_MImmigrating, toss\_MTempLeavingArea, toss\_NDeveloping, toss\_NDying, toss\_NMaturing, toss\_PDeveloping, toss\_PDying, toss\_PMaturing, toss\_PreparingForBreeding, toss\_ScaringOffChicks, toss\_StartingNewBrood, toss\_StoppingBreeding, tov\_Undefined, CfgInt::value(), CfgBool::value(), Skylark\_struct::x, and Skylark\_struct::y.

Referenced by Skylark\_Population\_Manager().

{

if ( cfg\_RipleysOutput\_used.value() ) {

OpenTheRipleysOutputProbe();

}

if ( cfg\_CIPEGridOutput\_used.value() ) {

OpenTheCIPEGridOutputProbe();

}

LoadParameters();

// autom. called by constructor

strcpy( m\_SimulationName, "Skylark" );

// Create 100 male and female skylarks

AdultSkylark\_struct \* aps;

aps = new AdultSkylark\_struct;

aps->size = 38;

aps->Terrs = TheSkylarkTerrs;

aps->L = m\_TheLandscape;

g\_land = m\_TheLandscape;

aps->sex = true;

for ( int i = 0; i < cfg\_SkStartNos.value(); i++ ) {

aps->x = random( m\_TheLandscape->SupplySimAreaWidth() );

aps->y = random( m\_TheLandscape->SupplySimAreaHeight() );

aps->bx = aps->x;

aps->by = aps->y;

aps->age = random( 5 );

aps->mh = tov\_Undefined;

CreateObjects( 3, NULL, NULL, aps, 1 );

}

aps->sex = false;

for ( int i = 0; i < cfg\_SkStartNos.value(); i++ ) {

aps->x = random( m\_TheLandscape->SupplySimAreaWidth() );

aps->y = random( m\_TheLandscape->SupplySimAreaHeight() );

aps->bx = aps->x;

aps->by = aps->y;

aps->age = random( 5 );

aps->mh = tov\_Undefined;

CreateObjects( 4, NULL, NULL, aps, 1 );

}

delete aps;

// Load List of Animal Classes

m\_ListNames[ 0 ] = "Clutch";

m\_ListNames[ 1 ] = "Nestling";

m\_ListNames[ 2 ] = "Pre-Fledgeling";

m\_ListNames[ 3 ] = "Male";

m\_ListNames[ 4 ] = "Female";

m\_ListNameLength = 5;

// Load State Names

StateNames[ toss\_Initiation ] = "Initiation";

//Clutch

StateNames[ toss\_Developing ] = "CDeveloping";

StateNames[ toss\_Hatching ] = "CHatching";

StateNames[ toss\_CDying ] = "CDying";

//Nestling

StateNames[ toss\_NDeveloping ] = "NDeveloping";

StateNames[ toss\_NMaturing ] = "NMaturing";

StateNames[ toss\_NDying ] = "NDying";

//PreFledgeling

StateNames[ toss\_PDeveloping ] = "PDeveloping";

StateNames[ toss\_PMaturing ] = "PMaturing";

StateNames[ toss\_PDying ] = "PDying";

//MaleSK

StateNames[ toss\_MFlocking ] = "MFlocking";

StateNames[ toss\_MFloating ] = "MFloating";

StateNames[ toss\_MArriving ] = "MArriving";

StateNames[ toss\_MImmigrating ] = "MImmigrating";

StateNames[ toss\_MEmigrating ] = "MEmigrating";

StateNames[ toss\_MTempLeavingArea ] = "MTempLeavingArea";

StateNames[ toss\_MFindingTerritory ] = "MFindingTerritory";

StateNames[ toss\_AttractingAMate ] = "MAttractingAMate";

StateNames[ toss\_FollowingMate ] = "MFollowingMate";

StateNames[ toss\_ScaringOffChicks ] = "MScaringOffChicks";

StateNames[ toss\_MCaringForYoung ] = "MCaringForYoung";

StateNames[ toss\_MDying ] = "MDying";

//FemaleSK

StateNames[ toss\_FFlocking ] = "FFlocking";

StateNames[ toss\_FFloating ] = "FFloating";

StateNames[ toss\_FArriving ] = "FArriving";

StateNames[ toss\_FImmigrating ] = "FImmigrating";

StateNames[ toss\_FEmigrating ] = "FEmigrating";

StateNames[ toss\_FTempLeavingArea ] = "FTempLeavingArea";

StateNames[ toss\_FFindingTerritory ] = "FFindingTerritory";

StateNames[ toss\_BuildingUpResources ] = "FBuildingUpResources";

StateNames[ toss\_MakingNest ] = "FMakingNest";

StateNames[ toss\_PreparingForBreeding ] = "FPreparingForBreeding";

StateNames[ toss\_Laying ] = "FLaying";

StateNames[ toss\_StartingNewBrood ] = "FStartingNewBrood";

StateNames[ toss\_EggHatching ] = "FEggHatching";

StateNames[ toss\_Incubating ] = "FIncubating";

StateNames[ toss\_StoppingBreeding ] = "FStoppingBreeding";

StateNames[ toss\_FCaringForYoung ] = "FCaringForYoung";

StateNames[ toss\_FDying ] = "FDying";

StateNames[ toss\_GivingUpTerritory ] = "FGivingUpTerritory";

StateNames[ toss\_Destroy ] = "Being Destroyed";

// determine whether we should shuffle, or sort or do nothing to skylarks

// after each time step.

BeforeStepActions[ 0 ] = 3; // 0 = Shuffle, 1 = SortX, 2 = SortY, 3 = do nothing

BeforeStepActions[ 1 ] = 3; // 0 = Shuffle, 1 = SortX, 2 = SortY, 3 = do nothing

BeforeStepActions[ 2 ] = 3; // 0 = Shuffle, 1 = SortX, 2 = SortY, 3 = do nothing

BeforeStepActions[ 3 ] = 0; // 0 = Shuffle, 1 = SortX, 2 = SortY, 3 = do nothing

BeforeStepActions[ 4 ] = 0; // 0 = Shuffle, 1 = SortX, 2 = SortY, 3 = do nothing

// Zero any counters

m\_TotalEggs = 0;

m\_TotalNestlings = 0;

m\_TotalPrefledgelings = 0;

m\_NoFledgeDeaths = 0;

m\_NoChickDeaths = 0;

m\_NoPestEffects = 0;

if ( cfg\_ReallyBigOutput\_used.value() ) {

OpenTheReallyBigProbe();

} else ReallyBigOutputPrb=0;

#ifdef \_\_SKPOM

SKPOM1 = fopen( "SkylarkPOM1.txt", "w" );

SKPOM2 = fopen( "SkylarkPOM2.txt", "w" );

#endif

}

|  |  |  |  |  |
| --- | --- | --- | --- | --- |
| bool Skylark\_Population\_Manager::IsBadWeather | ( |  | ) |  |

inline

{

return m\_IsBadWeather;

}

|  |  |  |  |  |  |
| --- | --- | --- | --- | --- | --- |
| void Skylark\_Population\_Manager::LoadParameters | ( | void |  | ) |  |

protected

References Breed\_Res\_Thresh1, ClutchMortProb, Cooling\_Rate\_Eggs, DensityScore, EggTemp, FemaleNestAcceptScore, FoodTripsPerDay, HeightScore, HQualityBareEarth, HQualityHedge, HQualityHedgeScrub, HQualityMetalRoad, HQualityNeutral, HQualityOpenTallVeg, HQualityTall, HQualityTall2, HQualityTallVeg, HQualityTrack, HQualityVeg30cm, HQualityWater, IsTramline, m\_NoChickDeaths, m\_NoFledgeDeaths, m\_NoPestEffects, MaxFeedRain, MD\_Hatch, MD\_Threshold, MeanExtractionRatePerMinute, MeanHatchingWeight, NestLeavingWeight, NestlingMortProb, NestPlacementMinQual, NotTramline, OpenTheBreedingSuccessProbe(), PatchyPremium, PreFledgeMortProb, RainHindrance, TempHindrance, CfgInt::value(), CfgFloat::value(), VegHindranceD, VegHindranceH, and VegTypeFledgelings.

Referenced by Init().

{

IsTramline = cfg\_tramline\_foraging.value();

NotTramline = 1 - IsTramline;

HQualityOpenTallVeg = cfg\_HQualityOpenTallVeg.value();

HQualityTall = cfg\_HQualityTall.value();

HQualityTall2 = cfg\_HQualityTall2.value();

HQualityTrack = cfg\_HQualityTrack.value();

HQualityMetalRoad = cfg\_HQualityMetalRoad.value();

HQualityHedgeScrub = cfg\_HQualityHedgeScrub.value();

HQualityNeutral = cfg\_HQualityNeutral.value();

HQualityHedge = cfg\_HQualityHedge.value();

HQualityTallVeg = cfg\_HQualityTallVeg.value();

HQualityWater = cfg\_HQualityWater.value();

HQualityVeg30cm = cfg\_HQualityVeg30cm.value();

HQualityBareEarth = cfg\_HQualityBareEarth.value();

PatchyPremium = cfg\_PatchyPremium.value();

FemaleNestAcceptScore = cfg\_FemaleMinTerritoryAcceptScore.value();

NestPlacementMinQual = cfg\_NestPlacementMinQual.value();

MaxFeedRain = cfg\_maxfeedrain.value(); //Estimate of really serious amount of rain

NestLeavingWeight = cfg\_NestLeavingWeight.value();

ClutchMortProb = cfg\_ClutchMortProb.value();

NestlingMortProb = cfg\_NestlingMortProb.value();

PreFledgeMortProb = cfg\_PreFledgeMortProb.value();

MeanHatchingWeight = cfg\_MeanHatchingWeight.value();

Cooling\_Rate\_Eggs = cfg\_Cooling\_Rate\_Eggs.value();

EggTemp = cfg\_EggTemp.value(); //was 37

MD\_Threshold = cfg\_MD\_Threshold.value(); // was 12

MeanExtractionRatePerMinute = cfg\_MeanExtractionRatePerMinute.value(); //Fitting parameter

FoodTripsPerDay = cfg\_FoodTripsPerDay.value(); // Poulsen

MD\_Hatch = cfg\_MinDaysToHatch.value() \* 24 \* 60 \* ( EggTemp - MD\_Threshold ); // 9.8 fastest development from field data

//MD\_Hatch = cfg\_MinDaysToHatch.value() \* 24 \* 60;

Breed\_Res\_Thresh1 = cfg\_Breed\_Res\_Thresh1.value();

for ( int h = 0; h <= 110; h++ ) {

DensityScore[ h ] = 0.0;

VegHindranceD[ h ] = 1.0;

HeightScore[ h ] = 0.0;

VegHindranceH[ h ] = 1.0;

}

// Vegetation hindrance is made up of two parts - a densithy and height part

// Each a linear after a threshold level, down to another threshold (zero)

double threshold = cfg\_heightconstant\_c.value();

for ( int h = (int) threshold; h <= 110; h++ ) {

VegHindranceH[ h ] = 1 + h \* cfg\_hindconstantH\_b.value();

if ( VegHindranceH[ h ] <= 0 ) VegHindranceH[ h ] = 0;

}

threshold = cfg\_densityconstant\_c.value();

for ( int d = (int) threshold; d <= 110; d++ ) {

VegHindranceD[ d ] =1 + d \* cfg\_hindconstantD\_b.value();

if ( VegHindranceD[ d ] <= 0 ) VegHindranceD[ d ] = 0;

}

// The next two arrays are related to nest choice

threshold = cfg\_densityconstant\_c.value();

for ( int h = (int) threshold; h <= 110; h++ ) {

// Power function

// DensityScore[ h ] = cfg\_densityconstant\_a.value() \* pow( h - ( threshold - 1 ), cfg\_densityconstant\_b.value() );

// Linear

DensityScore[ h ] = cfg\_densityconstant\_a.value() + (( h - threshold )\*( h - threshold ) \* cfg\_densityconstant\_b.value());

// Catch all just in case:

//if ( DensityScore[ h ] < 0 ) DensityScore[ h ] = 0;

}

threshold = cfg\_heightconstant\_c.value();

for ( int h = (int) threshold; h <= 110; h++ ) {

// Power function

//double hh = cfg\_heightconstant\_a.value() \* pow( h - ( threshold - 1 ), cfg\_heightconstant\_b.value() );

//HeightScore[ h ] = hh - ( 1 - hh ); // This expands the range of the curve

// Linear

HeightScore[ h ] = cfg\_heightconstant\_a.value() + ( h - threshold ) \* cfg\_heightconstant\_b.value();

// Catch all just in case:

//if ( HeightScore[ h ] < 0 ) HeightScore[ h ] = 0;

}

for ( int i = 0; i <= 30; i++ ) {

// TempHindrance[ i ] = (1-( pow( 30 - i, cfg\_temphindpow.value() ) / pow( 30, cfg\_temphindpow.value() ) ));

if ( i > 20 ) TempHindrance[ i ] = 1.0; else

TempHindrance[ i ] = 0.0

+ ( 1.0 \* ( 1 - ( pow( (double) 20 - i, (double) cfg\_temphindpow.value() ) / pow( (double) 20, (double) cfg\_temphindpow.value() ) ) ) );

}

for ( int i = 0; i <= 10; i++ ) {

RainHindrance[ i ] = pow( (double) 10 - i, (double) cfg\_rainhindpow.value() ) / (double) pow( (double) 10.0, (double) cfg\_rainhindpow.value() );

}

// Data for the FledgelingProbe

for ( int i = 0; i < 100; i++ ) VegTypeFledgelings[ i ] = 0;

m\_NoFledgeDeaths = 0;

m\_NoChickDeaths = 0;

m\_NoPestEffects = 0;

FILE \* MyFile = fopen( "PestEffects.txt", "w" );

fclose( MyFile );

OpenTheBreedingSuccessProbe();

fclose( MyFile );

}

|  |  |  |  |  |
| --- | --- | --- | --- | --- |
| bool Skylark\_Population\_Manager::OpenTheBreedingPairsProbe | ( |  | ) |  |

virtual

Reimplemented from Population\_Manager.

References g\_msg, MapErrorMsg::Warn(), and WARN\_FILE.

{

FILE \* MyFile = fopen("BreedingPairs.txt", "w" );

if ( !MyFile ) {

g\_msg->Warn( WARN\_FILE, "Skylark\_Population\_Manager::FledgelingProbeOutput():",

"Cannot open file: BreedingPairs.txt" );

exit( 0 );

}

fclose(MyFile);

return true;

}

|  |  |  |  |  |
| --- | --- | --- | --- | --- |
| bool Skylark\_Population\_Manager::OpenTheBreedingSuccessProbe | ( |  | ) |  |

virtual

Reimplemented from Population\_Manager.

References g\_msg, MapErrorMsg::Warn(), and WARN\_FILE.

Referenced by LoadParameters().

{

FILE \* MyFile = fopen("BreedingSuccess.txt", "w" );

if ( !MyFile ) {

g\_msg->Warn( WARN\_FILE, "Skylark\_Population\_Manager::FledgelingProbeOutput():",

"Cannot open file: BreedingSuccess.txt" );

exit( 0 );

}

fclose(MyFile);

return true;

}

|  |  |  |  |  |
| --- | --- | --- | --- | --- |
| bool Skylark\_Population\_Manager::OpenTheFledgelingProbe | ( |  | ) |  |

virtual

Reimplemented from Population\_Manager.

References Population\_Manager::m\_TheLandscape, tov\_Undefined, and Landscape::VegtypeToString().

{

FILE \* MyFile = fopen("SkFledgelingProbe.txt", "w" );

if ( !MyFile ) return false;

FILE \* MyFile2 = fopen("TOV\_KeyForFledgelingProbe.txt", "w" );

if ( !MyFile2 ) return false;

fprintf( MyFile2, "This is the tov to veg-type key\n" );

int howMany = ( int )tov\_Undefined;

for ( int i = 0; i <= howMany; i++ ) {

fprintf( MyFile2, "%d %s\n", i, m\_TheLandscape->VegtypeToString( (TTypesOfVegetation) i ) );

}

fclose( MyFile );

fclose( MyFile2 );

return false;

}

|  |  |  |  |
| --- | --- | --- | --- |
| float Skylark\_Population\_Manager::ProbePOM | ( | int | *ListIndex*, |
|  |  | probe\_data \* | *p\_TheProbe* |
|  | ) |  |  |

protected

Modified probe for POM Output.

References Skylark\_Male::HaveTerritory, probe\_data::m\_NoFarms, probe\_data::m\_RefFarms, Population\_Manager::TheArray, toss\_EggHatching, toss\_FCaringForYoung, toss\_Incubating, toss\_Laying, toss\_MakingNest, toss\_PreparingForBreeding, toss\_StartingNewBrood, and Skylark\_Base::WhatState().

Referenced by ProbeReportPOM().

{

// Counts through the list and goes through each area to see if the animal

// is standing there and if the farm conditions are met

float NumberSk = 0;

if (ListIndex==4) {

if ( p\_TheProbe->m\_NoFarms != 0 ) {

for ( unsigned j = 0; j < TheArray[ ListIndex ].size(); j++ ) {

unsigned Farm = TheArray[ ListIndex ] [ j ]->SupplyFarmOwnerRef();

for ( unsigned k = 0; k < p\_TheProbe->m\_NoFarms; k++ ) {

if ( p\_TheProbe->m\_RefFarms[ k ] == Farm ) {

Skylark\_Female \* FS;

FS = dynamic\_cast < Skylark\_Female \* > ( TheArray[ ListIndex ] [ j ] );

TTypesOfSkState state = (TTypesOfSkState) FS->WhatState();

switch (state) {

case toss\_MakingNest:

case toss\_PreparingForBreeding:

case toss\_Laying:

case toss\_StartingNewBrood:

case toss\_EggHatching:

case toss\_Incubating:

case toss\_FCaringForYoung:

NumberSk++; // it is in the square so increment number

default:

;

}

}

}

}

}

}

else if (ListIndex==3)

{

if ( p\_TheProbe->m\_NoFarms != 0 )

{

for ( unsigned j = 0; j < TheArray[ ListIndex ].size(); j++ )

{

unsigned Farm = TheArray[ ListIndex ] [ j ]->SupplyFarmOwnerRef();

for ( unsigned k = 0; k < p\_TheProbe->m\_NoFarms; k++ )

{

if ( p\_TheProbe->m\_RefFarms[ k ] == Farm )

{

Skylark\_Male \* MS;

MS = dynamic\_cast < Skylark\_Male \* > ( TheArray[ ListIndex ] [ j ] );

if (MS->HaveTerritory) NumberSk++;// it is in the square so increment number

}

}

}

}

}

return NumberSk;

}

|  |  |  |  |  |  |
| --- | --- | --- | --- | --- | --- |
| void Skylark\_Population\_Manager::ProbeReportPOM | ( | int | *a\_time* | ) |  |

protected

References probe\_data::FileOutput(), Population\_Manager::m\_NoProbes, ProbePOM(), Population\_Manager::SupplyListIndexSize(), and Population\_Manager::TheProbe.

Referenced by DoFirst().

{

for ( int ProbeNo = 0; ProbeNo < m\_NoProbes; ProbeNo++ ) {

int No = 0;

unsigned Index = SupplyListIndexSize();

for ( unsigned listindex = 0; listindex < Index; listindex++ ) {

if ( TheProbe[ ProbeNo ]->m\_TargetTypes[ listindex ] ) No += (int) ProbePOM( listindex, TheProbe[ ProbeNo ] );

}

TheProbe[ ProbeNo ]->FileOutput( No, a\_time, ProbeNo );

}

}

|  |  |  |  |  |  |
| --- | --- | --- | --- | --- | --- |
| void Skylark\_Population\_Manager::ReHouse | ( | void |  | ) |  |

protectedvirtual

References Skylark\_Male::HaveTerritory, Skylark\_Male::OnReHouse(), and Population\_Manager::TheArray.

{

Skylark\_Male\* MS = NULL; // assignment to get rid of warning

unsigned size = (unsigned) TheArray[ 3 ].size();

for ( unsigned j = 0; j < size; j++ ) {

MS = dynamic\_cast < Skylark\_Male \* > ( TheArray[ 3 ] [ j ] );

if (MS->HaveTerritory) {

MS->OnReHouse();

}

}

}

|  |  |  |  |  |  |
| --- | --- | --- | --- | --- | --- |
| void Skylark\_Population\_Manager::SetF\_Mig\_Mort | ( | int | *m* | ) |  |

inline

{

F\_Mig\_Mort = m;

}

|  |  |  |  |  |  |
| --- | --- | --- | --- | --- | --- |
| void Skylark\_Population\_Manager::SetM\_Mig\_Mort | ( | int | *m* | ) |  |

inline

{

M\_Mig\_Mort = m;

}

|  |  |  |  |  |
| --- | --- | --- | --- | --- |
| double Skylark\_Population\_Manager::SupplyEMi | ( |  | ) |  |

inline

Referenced by Skylark\_PreFledgeling::GetFledgelingEM(), Skylark\_PreFledgeling::GetFood(), Skylark\_Adult::RemoveEM(), and Skylark\_Female::st\_Incubating().

{

return EMi;

}

|  |  |  |  |  |
| --- | --- | --- | --- | --- |
| int Skylark\_Population\_Manager::SupplyF\_Mig\_Mort | ( |  | ) |  |

inline

{

return F\_Mig\_Mort;

}

|  |  |  |  |  |
| --- | --- | --- | --- | --- |
| int Skylark\_Population\_Manager::SupplyM\_Mig\_Mort | ( |  | ) |  |

inline

Referenced by Skylark\_Male::GetMigrationMortality().

{

return M\_Mig\_Mort;

}

|  |  |  |  |  |
| --- | --- | --- | --- | --- |
| int Skylark\_Population\_Manager::SupplyNoTerritories | ( |  | ) |  |

References SkTerritories::SupplyNoTerritories(), and TheSkylarkTerrs.

{

return TheSkylarkTerrs->SupplyNoTerritories();

}

|  |  |  |  |  |  |
| --- | --- | --- | --- | --- | --- |
| int Skylark\_Population\_Manager::TheBreedingFemalesProbe | ( | int | *ProbeNo* | ) |  |

virtual

Reimplemented from Population\_Manager.

References Skylark\_Male::HaveTerritory, Skylark\_Adult::Paired, and Population\_Manager::TheArray.

{

int NumberSk = 0;

Skylark\_Male \* MS;

for ( unsigned j = 0; j < TheArray[ 3 ].size(); j++ ) {

MS = dynamic\_cast < Skylark\_Male \* > ( TheArray[ 3 ] [ j ] );

/\* for (unsigned i=0; i<TheProbe[ProbeNo]->m\_NoAreas; i++) { if ((MS->m\_Born\_x>=(int)TheProbe[ProbeNo]->m\_Rect[i].m\_x1)

&& (MS->m\_Born\_y>=(int)TheProbe[ProbeNo]->m\_Rect[i].m\_y1) && (MS->m\_Born\_x<(int)TheProbe[ProbeNo]->m\_Rect[i].m\_x2)

&& (MS->m\_Born\_y<(int)TheProbe[ProbeNo]->m\_Rect[i].m\_y2) && (MS->Paired==true) && (MS->HaveTerritory==true))

NumberSk++; // it is in the square so increment number } \*/

if ( ( MS->Paired == true ) && ( MS->HaveTerritory == true ) )

NumberSk++; // it is in the square so increment number

}

return NumberSk;

}

|  |  |  |  |
| --- | --- | --- | --- |
| int Skylark\_Population\_Manager::TheBreedingSuccessProbe | ( | int & | *BreedingFemales*, |
|  |  | int & | *YoungOfTheYear*, |
|  |  | int & | *TotalPop*, |
|  |  | int & | *TotalFemales*, |
|  |  | int & | *TotalMales*, |
|  |  | int & | *BreedingAttempts* |
|  | ) |  |  |

virtual

Reimplemented from Population\_Manager.

References Skylark\_Female::ResetBreedingSuccess(), Skylark\_Female::Supply\_BreedingAttempts(), Skylark\_Female::Supply\_BreedingSuccess(), Population\_Manager::TheArray, and TheFledgelingProbe().

{

int NumberA = 0;

int HowMany = 0;

BreedingAttempts = 0;

Skylark\_Female \* FS;

unsigned sz = (int)TheArray[ 4 ].size();

for ( unsigned j = 0; j < sz; j++ ) {

FS = dynamic\_cast < Skylark\_Female \* > ( TheArray[ 4 ] [ j ] );

int success = FS->Supply\_BreedingSuccess();

#ifdef \_\_HazelParry1

if ( success >0 ) {

NumberA += success;

HowMany++;

FS->ResetBreedingSuccess();

}

#else

if ( success != -1 ) {

NumberA += success;

HowMany++;

}

#endif

BreedingAttempts += FS->Supply\_BreedingAttempts();

}

BreedingFemales = HowMany;

TotalFemales = sz;

TotalMales = (int)TheArray[ 3 ].size();

YoungOfTheYear = TheFledgelingProbe();

TotalPop = TotalFemales + TotalMales;

return NumberA;

}

|  |  |  |  |  |
| --- | --- | --- | --- | --- |
| void Skylark\_Population\_Manager::TheCIPEGridOutputProbe | ( |  | ) |  |

virtual

This method MUST be overridden in descendent classes, if you want it to work

Reimplemented from Population\_Manager.

References Population\_Manager::CIPEGridOutputPrb, Population\_Manager::m\_cgridcount, Population\_Manager::m\_cgridcountwidth, Population\_Manager::m\_cipegridsize, Skylark\_Adult::Paired, sob\_Female, TAnimal::Supply\_m\_Location\_x(), TAnimal::Supply\_m\_Location\_y(), and Population\_Manager::TheArray.

{

Skylark\_Female\* FS;

unsigned totalF=(int)TheArray[sob\_Female].size();

int x,y;

int totalcells = m\_cgridcountwidth\*m\_cgridcountwidth;

for (int i=0; i<totalcells; i++) m\_cgridcount[i] = 0;

for (unsigned j=0; j<totalF; j++)

{

FS=dynamic\_cast<Skylark\_Female\*>(TheArray[sob\_Female][j]);

if (FS->Paired) {

x=FS->Supply\_m\_Location\_x();

y=FS->Supply\_m\_Location\_y();

int gx=x/m\_cipegridsize; // 500m grids is default

int gy=y/m\_cipegridsize;

m\_cgridcount[gx+gy\*m\_cgridcountwidth]++;

}

}

for (int i=0; i<totalcells; i++) fprintf(CIPEGridOutputPrb,"%d\t",m\_cgridcount[i]);

fprintf(CIPEGridOutputPrb,"\n");

fflush(CIPEGridOutputPrb);

}

|  |  |  |  |  |
| --- | --- | --- | --- | --- |
| int Skylark\_Population\_Manager::TheFledgelingProbe | ( |  | ) |  |

virtual

Reimplemented from Population\_Manager.

References Skylark\_Base::Age, Skylark\_Base::m\_MyHome, Population\_Manager::TheArray, and VegTypeFledgelings.

Referenced by TheBreedingSuccessProbe().

{

int NumberSk = 0;

Skylark\_Male \* MS;

for ( unsigned j = 0; j < TheArray[ 3 ].size(); j++ ) {

MS = dynamic\_cast < Skylark\_Male \* > ( TheArray[ 3 ] [ j ] );

if ( MS->Age == 0 ) {

NumberSk++; // it is in the square so increment number

VegTypeFledgelings[ MS->m\_MyHome ] ++;

}

}

Skylark\_Female \* FS;

for ( unsigned j = 0; j < TheArray[ 4 ].size(); j++ ) {

FS = dynamic\_cast < Skylark\_Female \* > ( TheArray[ 4 ] [ j ] );

if ( FS->Age == 0 ) {

NumberSk++; // it is in the square so increment number

VegTypeFledgelings[ FS->m\_MyHome ] ++;

}

}

return NumberSk;

}

|  |  |  |  |  |
| --- | --- | --- | --- | --- |
| void Skylark\_Population\_Manager::TheNWordOutputProbe | ( |  | ) |  |

virtual

Output file facility added in November 2009

Reimplemented from Population\_Manager.

References Population\_Manager::m\_gridcount, Population\_Manager::m\_gridcountsize, Population\_Manager::m\_gridcountwidth, Population\_Manager::NWordOutput(), Skylark\_Adult::Paired, sob\_Female, TAnimal::Supply\_m\_Location\_x(), TAnimal::Supply\_m\_Location\_y(), and Population\_Manager::TheArray.

{

Skylark\_Female\* FS;

unsigned int totalF= (unsigned int) TheArray[sob\_Female].size();

int x,y;

int totalcells[4];

for (int grid=0; grid<4; grid++) {

totalcells[grid] = m\_gridcountwidth[grid]\*m\_gridcountwidth[grid];

for (int i=0; i<totalcells[grid]; i++) m\_gridcount[grid][i] = 0;

for (unsigned j=0; j<totalF; j++) //adult females

{

FS=dynamic\_cast<Skylark\_Female\*>(TheArray[sob\_Female][j]);

if (FS->Paired) {

x=FS->Supply\_m\_Location\_x();

y=FS->Supply\_m\_Location\_y();

int gx=x/m\_gridcountsize[grid]; // 500m grids is default

int gy=y/m\_gridcountsize[grid];

m\_gridcount[grid][gx+gy\*m\_gridcountwidth[grid]]++;

}

}

}

NWordOutput();

}

|  |  |  |  |  |  |
| --- | --- | --- | --- | --- | --- |
| void Skylark\_Population\_Manager::TheRipleysOutputProbe | ( | FILE \* | *a\_prb* | ) |  |

virtual

This method must be overridden in descendent classes

Reimplemented from Population\_Manager.

References Population\_Manager::m\_TheLandscape, Skylark\_Adult::Paired, TAnimal::Supply\_m\_Location\_x(), TAnimal::Supply\_m\_Location\_y(), Landscape::SupplySimAreaWidth(), and Population\_Manager::TheArray.

{

Skylark\_Female\* FS;

unsigned totalF=0;

for (unsigned j=0; j<TheArray[4].size(); j++) //adult females

{

FS=dynamic\_cast<Skylark\_Female\*>(TheArray[4][j]);

if (FS->Paired) totalF++;

}

int x,y;

int w = m\_TheLandscape->SupplySimAreaWidth();

int h = m\_TheLandscape->SupplySimAreaWidth();

fprintf(a\_prb,"%d %d %d %d %d\n", 0,w ,0, h, totalF);

for (unsigned j=0; j<TheArray[4].size(); j++) //adult females

{

FS=dynamic\_cast<Skylark\_Female\*>(TheArray[4][j]);

if (FS->Paired) {

x=FS->Supply\_m\_Location\_x();

y=FS->Supply\_m\_Location\_y();

fprintf(a\_prb,"%d\t%d\n", x,y);

}

}

fflush(a\_prb);

}

|  |  |  |  |  |  |
| --- | --- | --- | --- | --- | --- |
| int Skylark\_Population\_Manager::TheSkylarkTerrsSupply\_quality | ( | int | *r* | ) |  |

References SkTerritories::Supply\_quality(), and TheSkylarkTerrs.

{

return TheSkylarkTerrs->Supply\_quality( r );

}

|  |  |  |  |  |  |
| --- | --- | --- | --- | --- | --- |
| int Skylark\_Population\_Manager::TheSkylarkTerrsSupply\_size | ( | int | *r* | ) |  |

References SkTerritories::Supply\_size(), and TheSkylarkTerrs.

{

return TheSkylarkTerrs->Supply\_size( r );

}

|  |  |  |  |  |  |
| --- | --- | --- | --- | --- | --- |
| int Skylark\_Population\_Manager::TheSkylarkTerrsSupply\_x | ( | int | *r* | ) |  |

References SkTerritories::Supply\_x(), and TheSkylarkTerrs.

{

return TheSkylarkTerrs->Supply\_x( r );

}

|  |  |  |  |  |  |
| --- | --- | --- | --- | --- | --- |
| int Skylark\_Population\_Manager::TheSkylarkTerrsSupply\_y | ( | int | *r* | ) |  |

References SkTerritories::Supply\_y(), and TheSkylarkTerrs.

{

return TheSkylarkTerrs->Supply\_y( r );

}

|  |  |  |  |
| --- | --- | --- | --- |
| void Skylark\_Population\_Manager::WriteSKPOM1 | ( | int | *n*, |
|  |  | int | *n2* |
|  | ) |  |  |

inline

Referenced by Skylark\_Female::OnMateDying(), Skylark\_Female::OnMateHomeless(), Skylark\_Clutch::st\_Developing(), Skylark\_Female::st\_Dying(), Skylark\_Female::st\_GivingUpTerritory(), Skylark\_Female::st\_StartingNewBrood(), Skylark\_Female::st\_StoppingBreeding(), and Skylark\_Female::Step().

{

fprintf( SKPOM1, "%i\t%i\n", n, n2 );

}

|  |  |  |  |
| --- | --- | --- | --- |
| void Skylark\_Population\_Manager::WriteSKPOM2 | ( | int | *n*, |
|  |  | int | *n2* |
|  | ) |  |  |

inline

Referenced by Skylark\_Nestling::OnDeserted(), Skylark\_Nestling::OnYouHaveBeenEaten(), and Skylark\_Nestling::st\_Developing().

{

fprintf( SKPOM2, "%i\t%i\n", n, n2 );

}

---

## Member Data Documentation

double Skylark\_Population\_Manager::EMi

protected

Referenced by DoFirst().

int Skylark\_Population\_Manager::F\_Mig\_Mort

protected

Referenced by DoFirst(), and Skylark\_Population\_Manager().

bool Skylark\_Population\_Manager::m\_IsBadWeather

protected

Referenced by DoFirst().

int Skylark\_Population\_Manager::M\_Mig\_Mort

protected

Referenced by DoFirst(), and Skylark\_Population\_Manager().

int Skylark\_Population\_Manager::m\_NoChickDeaths

protected

Referenced by DoFirst(), Init(), and LoadParameters().

int Skylark\_Population\_Manager::m\_NoFledgeDeaths

protected

Referenced by DoFirst(), Init(), and LoadParameters().

int Skylark\_Population\_Manager::m\_NoPestEffects

protected

Referenced by DoFirst(), Init(), and LoadParameters().

int Skylark\_Population\_Manager::m\_StriglingMort[4]

protected

Referenced by BreedingSuccessProbeOutput(), and DoFirst().

int Skylark\_Population\_Manager::m\_TotalEggs

protected

Referenced by BreedingSuccessProbeOutput(), and Init().

int Skylark\_Population\_Manager::m\_TotalNestlings

protected

Referenced by BreedingSuccessProbeOutput(), CreateObjects(), and Init().

int Skylark\_Population\_Manager::m\_TotalPrefledgelings

protected

Referenced by BreedingSuccessProbeOutput(), CreateObjects(), and Init().

FILE\* Skylark\_Population\_Manager::SKPOM1

protected

Referenced by Init(), and ~Skylark\_Population\_Manager().

FILE\* Skylark\_Population\_Manager::SKPOM2

protected

Referenced by Init(), and ~Skylark\_Population\_Manager().

SkTerritories\* Skylark\_Population\_Manager::TheSkylarkTerrs

Referenced by DoFirst(), Init(), Skylark\_Male::OnEvicted(), Skylark\_Population\_Manager(), Skylark\_Male::st\_Arriving(), SupplyNoTerritories(), TheSkylarkTerrsSupply\_quality(), TheSkylarkTerrsSupply\_size(), TheSkylarkTerrsSupply\_x(), TheSkylarkTerrsSupply\_y(), and ~Skylark\_Population\_Manager().

int Skylark\_Population\_Manager::VegTypeFledgelings[100]

Referenced by FledgelingProbeOutput(), LoadParameters(), and TheFledgelingProbe().

---

The documentation for this class was generated from the following files:

- skylarks\_all.h
- skylarks\_all.cpp


- Skylark\_Population\_Manager
- Generated on Thu Jan 10 2013 13:15:38 for ALMaSS Skylark ODdox by
   1.8.1.1
